# Single-nucleus RNA sequencing and lipidomics reveal characteristics of transcriptional and lipid composition in porcine *longissimus dorsi* muscle

**DOI:** 10.1186/s12864-024-10488-8

**Published:** 2024-06-20

**Authors:** Lanlan Yi, Qiuyan Li, Junhong Zhu, Wenjie Cheng, Yuxiao Xie, Ying Huang, Hongye Zhao, Meilin Hao, Hongjiang Wei, Sumei Zhao

**Affiliations:** 1 Yunnan Key Laboratory of Animal Nutrition and Feed Science, Yunnan Agricultural University, Kunming, 650201 Yunnan China; 2 College of Biology and Agriculture (College of Food Science and Technology), Zunyi Normal College, Zunyi, 563006 China; 3 Yunnan Province Key Laboratory for Porcine Gene Editing and Xenotransplantation, Yunnan Agricultural University, Kunming, 650201 China

**Keywords:** Pigs, *Longissimus dorsi* muscle, Intramuscular fat, snRNA-seq, Lipidomics

## Abstract

**Background:**

Global per capita meat consumption continues to rise, especially pork. Meat quality is influenced by the content of intramuscular fat (IMF) as a key factor. The *longissimus dorsi* muscle of Dahe pigs (DHM, IMF: 7.98% ± 1.96%) and Dahe black pigs (DHBM, IMF: 3.30% ± 0.64%) was studied to explore cellular heterogeneity and differentially expressed genes (DEGs) associated with IMF deposition using single-nucleus RNA sequencing (snRNA-seq). The lipid composition was then analyzed using non-targeted lipidomics.

**Results:**

A total of seven cell subpopulations were identified, including myocytes, fibroblast/fibro/adipogenic progenitors (FAPs), satellite cells, endothelial cells, macrophages, pericytes, and adipocytes. Among them, FAPs and adipocytes were more focused because they could be associated with lipid deposition. 1623 DEGs in the FAPs subpopulation of DHBM were up-regulated compared with DHM, while 1535 were down-regulated. These DEGs enriched in the glycolysis/gluconeogenesis pathway. 109 DEGs were up-regulated and 806 were down-regulated in the adipocyte subpopulation of DHBM compared with DHM, which were mainly enriched in the PPAR signaling pathway and fatty acid (FA) biosynthesis. The expression level of *PPARG*, *ABP4*, *LEP*, and *ACSL1* genes in DHM was higher than that in DHBM. Lipidomics reveals porcine lipid composition characteristics of muscle tissue. A total of 41 lipid classes and 2699 lipid species were identified in DHM and DHBM groups. The top ten relative peak areas of lipid classes in DHM and DHBM were triglyceride (TG), phosphatidylcholine (PC), phosphatidylethanolamine (PE), phosphatidylserine (PS), diglyceride (DG), cardiolipin (CL), ceramides (Cer), Simple Glc series (Hex1Cer), sphingomyelin (phSM), and phosphatidylinositol (PI). The relative peak areas of 35 lipid species in DHM were lower than DHBM, and 28 lipid species that were higher. There was a significant increase in the TG fatty acyl chains C6:0, C17:0, and C11:4, and a significant decrease in C16:0, C18:1, C18:2, and C22:4 in DHBM (*p* < 0.05).

**Conclusions:**

C16:0 FA may downregulate the expression level of *PPARG* gene, which leads to the downregulation of fat metabolism-related genes such as *ACSL*, *PLIN2*, and *FABP4* in DHBM compared with DHM. This may be the reason that the lipid deposition ability of Dahe pigs is stronger than that of Dahe black pigs, which need further investigation.

**Supplementary Information:**

The online version contains supplementary material available at 10.1186/s12864-024-10488-8.

## Background

Pork is the most widely consumed meat in the world and plays a crucial role in ensuring global food security [1, 2]. The deposition and composition of lipids in pork affect its sensory and nutritional quality, with triglycerides (TG) being the main form of lipid storage [3]. Muscle energy metabolism regulates an organism's energy consumption and fat deposition, affecting intramuscular fat content (IMF), muscle fiber type, meat color, tenderness, pH value, and drip loss [4]. IMF refers to the chemically extractable fat in muscle samples, primarily from adipocytes and muscle cells, which increases mainly due to the increase in TG content [5]. IMF content and composition are the main factor attributes to meat quality [6]. In the era of omics, based on high-throughput sequencing, research on gene expression in domestic animals has exploded. Since 2001, numerous genes associated with the regulation of lipogenesis and IMF deposition have been identified in transcriptomic studies of porcine IMF deposition [6].

The differentially expressed genes (DEGs) in the transcriptome results of the *longissimus dorsi* muscle of Northeast Min pigs and Changbaishan wild boars are mainly associated with muscle fiber development, differentiation, and growth, as well as with lipogenesis and lipolysis in skeletal muscle [7]. Iberian pork is known for its high quality and elevated IMF content, and the transcriptomic analysis of the *longissimus dorsi* muscle revealed high expression of genes involved in regulating lipid metabolism [8]. Many DEGs in the skeletal muscle of Shaziling pigs and Yorkshire pigs are associated with lipid mobilization, energy metabolism, cytoskeleton, and signal transduction [9]. The DEGs that were up-regulated in Yorkshire pigs mainly involve glucose metabolism, glycolysis, and muscle development, while the up-regulated DEGs in Wannan pigs are associated with myofibrils, fatty acid (FA) metabolism, and lipid catabolism [10]. It is generally believed that the IMF content of China's native pig breeds is higher than that of traditional lean pig breeds such as Landrace, Yorkshire, and Duroc pigs [11–14]. However, muscle tissue contains a variety of cell types, and transcriptomics can only capture the average expression levels of the tissue, making it difficult to elucidate the variations between individual cells.

In 2009, Tang et al. [15] published a paper on single cell transcriptome sequencing (scRNA-seq) technology. The minimum functional unit of skeletal muscle is the myofiber, which is often longer than 100 µm, exceeding the 30 µm cell size limit for single-cell analysis [16]. Although there are specific biases between single-nucleus RNA sequencing (snRNA-seq) and scRNA-seq methods, similar cell clustering results can generally be obtained [17, 18]. Skeletal muscles are composed of various cell types, including myocytes, mesenchymal stem cells, endothelial cells, smooth muscle cells, muscle satellite cells, neuronal cells, and immune cells [19]. There have been many studies on the application of scRNA-seq in human and mouse skeletal muscles. However, there are few studies on the heterogeneity of porcine muscle tissue.

The Dahe pig is a traditional local breed in China with a long history of breeding. Dahe Black Pig is a hybrid breed resulting from a crossbreeding program between the Dahe Pig and Duroc, which have been detailed the snRNA-seq profile of the livers in our previous work [20]. It was officially recognized as a new breed in 2003. Fat is deposited in muscle tissue, so the differences in lipid metabolism in the muscles of these two breeds were studied. In this study, the Dahe pigs and Dahe black pigs were used as experimental subjects to explore the mechanism of IMF differences in the *longissimus dorsi* muscle using single-nucleus transcriptome and lipidomics. The study results will provide new insights into the cell types of the longissimus dorsi muscle in Dahe pigs and Dahe black pigs, the DEGs involved in signaling pathway, and the lipid composition.

## Methods

### Animals

All animal procedures followed the animal care guidelines approved by the Animal Care and Use Committee of Yunnan Agricultural University (Approval Code: 202,105,017). Dahe and Dahe Black pigs were providing from the Dahe Black Pig Research Institute of Fuyuan County. Six boars per breed were selected and fed the same basic diet. Twelve pigs, at 194 days of age, were stunned with electric shock before being slaughtered. Samples of the *longissimus dorsi* muscle for snRNA-seq and lipidomics were then collected, frozen in liquid nitrogen, and stored at -80 °C. *Longissimus dorsi* muscle samples for chemical composition determination were dehydrated immediately after collection.

### The chemical composition of porcine *longissimus dorsi* muscle

The chemical composition of the completely dehydrated *longissimus dorsi* muscle samples was determined according to Chinese national standards. The Soxhlet extraction method was used to determine the ether extracts (EE) in the samples (GB5009.6–2016 [21]). The EE content in the sample is calculated as the weight of fat extracted with petroleum ether divided by the mass of the sample. The boiling point of petroleum ether used in this experiment is 30–60 °C. The Kjeldahl method was used to determine the crude protein (CP) content in the sample (GB5009.5–2016 [22]). The volume of HCl standard titration solution consumed by the sample decomposition solution is multiplied by the HCl concentration and then by 0.014, and the result is multiplied by 6.25 and divided by the sample mass to obtain the protein mass fraction. To measure the ash content, the sample must first be burned to carbonize (GB5009.4–2016 [23]). The sample that was carbonized to be smokeless was placed in a high-temperature furnace and heated to 550 °C for 4 h until no carbon particles remained. The mass of the sample after ignition divided by the initial mass of the sample gives the ash content of the sample. EE, CP and Ash are the results of the determination of dehydrated meat samples. These results were further converted to the content in a fresh meat sample of the same mass as the dehydrated meat sample.

### snRNA-seq using 10 × genomics chromium

#### snRNA-seq library preparation and sequencing

Take approximately 0.2 g of porcine *longissimus dorsi* muscle sample, place it in a sterile petri dish, and finely chop the tissue on ice into pieces measuring 1–2 mm^3^ in size. Transfer the tissue to a Dounce homogenizer and add 500 μL of pre-cooled lysis buffer (0.25 M sucrose, 5 mM CaCl_2_, 3 mM MgAc_2_, 10 mM Tris–HCl pH 8.0, 1 mM DTT, 0.1 mM EDTA, 1 × protease inhibitor, 1 U/μL RiboLock RNase Inhibitor) into the tissue sample tube. Submerge the tissue and wait for it to thaw. Grind the tissue into a homogenate, add 700 μL of nuclear washing buffer (PBS containing 0.04% BSA, 0.2 U/μL RiboLock RNase Inhibitor, 500 mM mannitol, 0.1 mM PMSF Protease Inhibitor), and mix by inversion. Filter the tissue homogenate through a 70 μm cell sieve to obtain approximately 1 mL of filtrate. Add 1 mL of 50% iodixanol (0.16 M sucrose, 10 mM NaCl, 3 mM MgCl_2_, 10 mM Tris–HCl pH 7.4, 1 U/μL RiboLock RNase Inhibitor, 1 mM DTT, 0.1 mM PMSF Protease Inhibitor) to the filtrate. Obtain an iodixanol solution containing cell nuclei. Prepare a gradient solution by combining 1 mL of 33% iodixanol with 2 mL of 30% iodixanol. Next, add 2 mL of iodixanol solution containing cell nuclei, and centrifuge at 10,000 × g for 20 min at 4 °C. There will be a white nuclear layer at the interface between a 33% iodixanol solution and a 30% iodixanol solution. Keep the nuclear layer intact and resuspend the nuclei in nucleus wash buffer. Filter the nuclei suspension using a 40 μm filter. Centrifuge at 500 × g for 5 min at 4 °C, transfer the supernatant to a new tube, and add 100 μL of nuclear lysis solution to resuspend the nuclear pellet. Utilize a microscope and a cell counting board to identify the nuclear suspension following trypan blue staining. Then, determine the total number of nuclei, concentration, and the percentage of nuclei with intact nuclear membranes. The machine can label qualified cell nucleus suspensions. The target concentration of the cell nucleus suspension for labeling is 700–1200 nucleus/µL.

### Cell clustering and marker gene identification

Gel Bead-In-EMlusion (GEM) was generated from the nuclear suspension using the 10X Genomics GemCode Single-cell instrument. Libraries were generated and sequenced using Chromium Next GEM Single Cell 3’Reagent Kits v3.1. After the gel beads are dissolved in GEM, primers containing the Illumina® R1 sequence, 16 nt 10 × barcode, 10 nt unique molecular identifier (UMI), and release poly-dT primer sequences are mixed with cell lysate and master mix. The barcoded full-length cDNA is then reverse transcribed from the polyadenylated mRNA. Use silane magnetic beads to remove any remaining biochemical reagents and primers from the reaction mixture following GEM. The barcoded full-length cDNA was then amplified by PCR.

The 10X Genomics Cell Ranger software (version 3.1.0) was utilized to convert raw BCL files to FASTQ files, perform alignment, and quantify counts. Before quantification, correct UMI sequencing errors by identifying valid barcodes based on the EmptyDrops method [24]. Nuclei with an unusually high number of UMIs (≥ 8000) were filtered out. Nuclei with fewer than 500 or more than 4000 detected genes were excluded. Additionally, DoubletFinder (version 2.0.3) was used to filter bimodal GEMs [25]. After removing unwanted cells from the dataset, gene expression measurements for each cell were normalized to total expression. The gene expression matrix of each cellular sample was individually imported into Seurat [26] version 3.1.1 for subsequent analysis.

The integrated expression matrix was then scaled and subjected to principal component analysis to reduce dimensionality. Uniform manifold approximation and projection (UMAP) uses Euclidean distance calculation to measure the distance between subgroups. Cell types are annotated according to markers identified in the literature. Use the ggplot2 package (version 2.3.2.1) to generate bubble charts for marker genes and heat maps for the top 20 DEGs. The cells were analyzed over time using the monocle2 package (Version 2.10.1). Cells are arranged along a cell trajectory based on pseudotime changes, simulating the cell differentiation relationship during development.

The expression value of each gene in a given cluster was compared with the remaining cells using the Wilcoxon rank sum test (*p* < 0.05) [27]. The gene is expressed in more than 25% of the cells in the target cluster. Overexpressed in the target cluster by at least 1.28-fold. Genes that meet the above conditions are considered DEGs.

### Functional enrichment analysis of DEGs in cell subpopulations

DEGs between DHM and DHBM were used for functional annotation. The basic unit of the Gene Ontology (GO) is the GO term. Each GO term can be assigned to a molecular function, cellular component, or biological process [28]. Screen DEGs associated with specific biological functions. All genes related to peaks were mapped to GO terms in the GO database (http://www.geneontology.org/), and the gene count for each term was calculated. Significantly enriched GO terms among DEGs were determined using a hypergeometric test (*p* < 0.05). Kyoto Encyclopedia of Genes and Genomes (KEGG) is a major public pathway-related database [29] that can connect genes and biological functions. DEGs were enriched into pathways. Use the ggplot2 package (version 2.3.2.1) to create GO bubble charts and KEGG bar charts. The single nucleus RNA sequencing data used in this study is deposited in NCBI databases under accession number: PRJNA1113324.

### Untargeted lipidomics of porcine *longissimus dorsi* muscle

#### Lipid extraction of samples

Lipids were extracted using the methyl tert-butyl ether (MTBE) method. Add the sample and homogenize it with 200 µL of water and 240 µL of methanol. Then add 800 µL of MTBE, sonicate at 4 °C for 20 min, and let it stand at room temperature for 30 min. The solution was centrifuged at 10 °C and 14,000 g for 15 min to separate the upper organic solvent layer, which was then dried under nitrogen.

### LC–MS method for lipid analysis

Reverse phase chromatography was selected for LC separation using CSH C18 column (1.7 µm, 2.1 mm × 100 mm, Waters). The lipid extracts were re-dissolved in 200 µL 90% isopropanol/acetonitrile, centrifuged at 14,000 g for 15 min, finally 3 µL of sample was injected. Solvent A was acetonitrile–water (6:4, v/v) with 0.1% formic acid and 0.1 mM ammonium formatted and solvent B was acetonitrile-isopropanol (1:9, v/v) with 0.1% formic acid and 0.1 mM ammonium formatted. The initial mobile phase was 40% solvent B at a flow rate of 300 μL/min. It was held for 3.5 min, and then linearly increased to 75% solvent B in 9.5 min, and then linearly increased to 99% solvent B in 6 min, followed by equilibrating at 40% solvent B for 5 min. Mass spectra was acquired by Q-Exactive Plus in positive and negative mode, respectively. ESI parameters were optimized and preset for all measurements as follows: Source temperature, 300 °C; Capillary Temp, 350 °C, the ion spray voltage was set at 3000 V, S-Lens RF Level was set at 50% and the scan range of the instruments was set at m/z 200–1800.

### Lipid identification and correlation analysis of differential lipid species

The raw data were converted to mz.XML format using ProteoWizard, and then XCMS software was employed for peak alignment, retention time correction, and peak area extraction. Use Lipid Search to identify different lipid classes. This database contains more than 30 lipid classes and over 1,500,000 fragment ions. Mass tolerances were set at 5 ppm for both precursors and fragments. Use GraphPad Prism 8 to create bar charts representing lipid numbers, pie charts and histograms representing lipid class. Partial Least Squares Discriminant Analysis (PLS-DA) is a supervised statistical method for discriminant analysis. To prevent overfitting of the supervised model during the modeling process, the permutation test is employed to validate the model's accuracy. Pearson correlation analysis was conducted to assess the correlation between samples and differential lipid species, as well as between differential lipid species.

### Data analysis

The materials in the solution flow chart come from BioRender and are drawn using drow.io software. All statistical analyses of the lipidomic data described in this work were calculated based on relative abundance. Significance testing between groups was conducted using a t-test. Differences between groups were deemed statistically significant (*P* < 0.05). Spearman correlation analysis was performed to determine the correlation between the DEGs related to lipogenesis in adipocytes and the differential lipid species classified as TG and phosphatidylinositols (PI).

## Results

### Chemical composition characteristics of porcine *longissimus dorsi* muscle

**Fig. 1:**
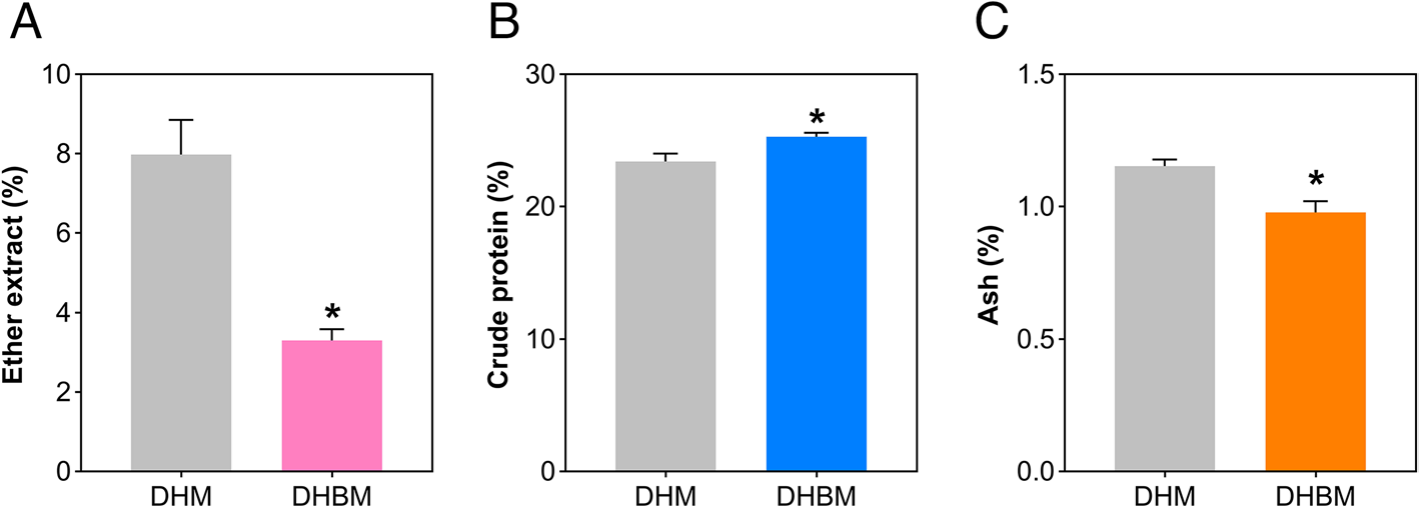
Chemical composition of *longissimus dorsi* muscle from Dahe pigs and Dahe Black pigs. * means significantly different at *p* < 0.05

The chemical composition of *longissimus dorsi* muscle from Dahe pigs and Dahe Black pigs is showed in Fig. 1. Significant differences between the two breeds were observed in EE, protein and ash (*p* < 0.05). There were decreased EE (Fig. 1A) and ash (Fig. 1C) level in DHM and DHBM. The EE and ash contents of the *longissimus dorsi* muscle of DHBM were 3.30% and 0.98% respectively, which were significantly lower than those of DHM (7.98% and 1.15%) (Additional file 1). The CP content of DHBM is 25.29%, which is significantly higher than DHM (23.41%) (Fig. 1B, Additional file 1).

### snRNA-seq identified distinct cell populations in porcine *longissimus dorsi* muscle

**Fig. 2:**
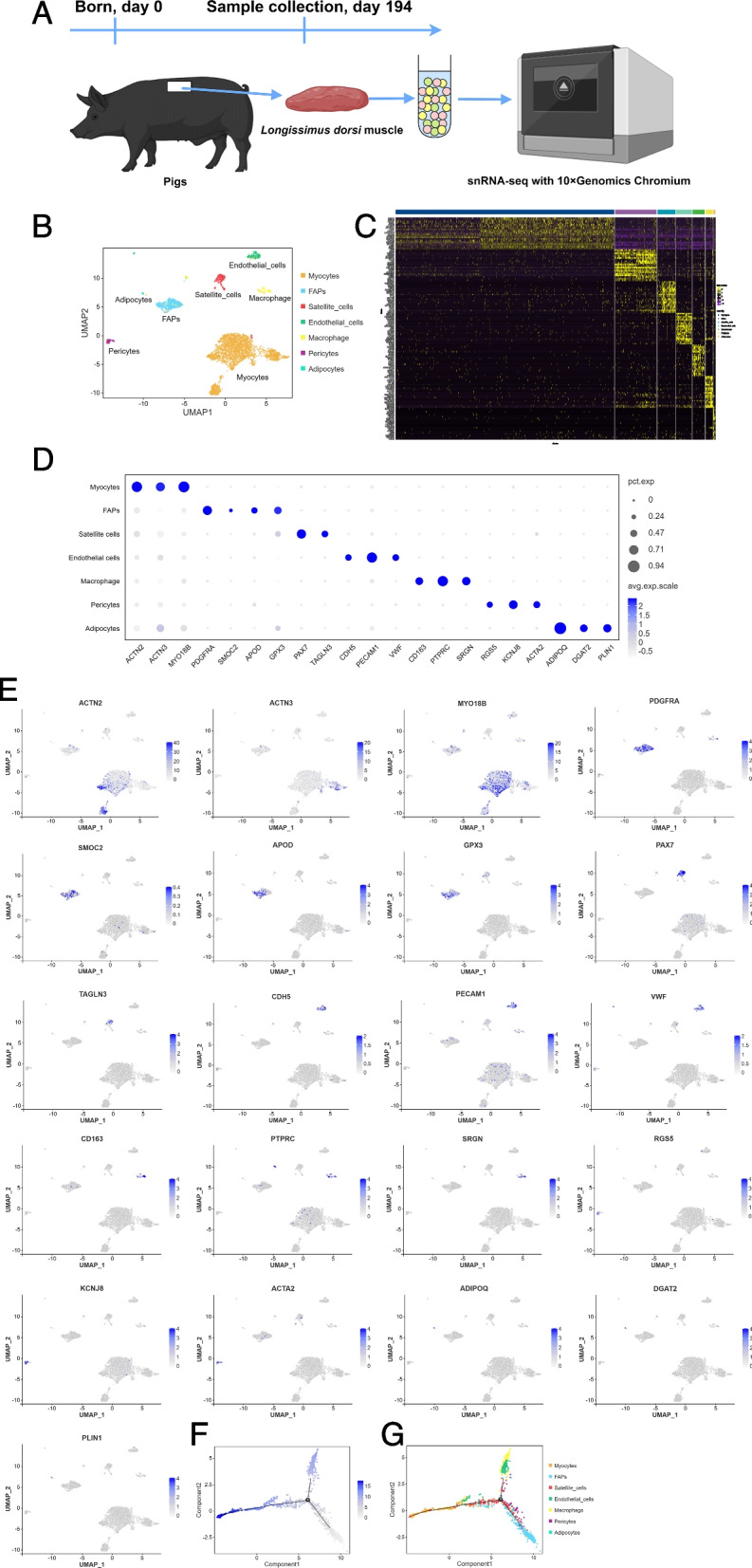
snRNA-seq identifies different cell populations in porcine *longissimus dorsi* muscle. **A** Protocol for preparing the *longissimus dorsi* muscle from Dahe pigs and Dahe Black pigs and using it for snRNA-seq. **B** cell type UMAP plot of *longissimus dorsi* muscle from two different breeds. The cell color is determined by the cell type, which is associated with different marker genes. **C** Heatmap of the 20 most DEGs in each of the seven identified cell types. **D** Dot plot of gene expression of cell-type differentiating markers. The size of the dot represents the expression ratio of the gene in the cell type, and the color represents the normalized value of the average expression level of the cell type. **E** Marker gene expression used to identify cell type in integrated UMAP. **F** Pseudotemporal differentiation state of cells. The lighter the color, the earlier the development period. **G** Pseudotime ordering of all cell types. Dots of the same color represent the same cell type, and each branch represents a cell state

Single-nucleated cells from *longissimus dorsi* muscle samples of 194-day-old Dahe pig and Dahe Black pig were used for snRNA-seq by 10 × Gnomics (Fig. 2A). Details such as sequencing reads, cell numbers, and gene numbers are shown in Additional file 2. Dahe Black pig has 1890 cells for downstream analysis. The mean reads per cell is 193,574, and the median UMI counts per cell is 4138. A total of 22,312 genes were detected. Using unsupervised clustering, seven cell types were resolved (Fig. 2B). The visualization of the top 20 most variably expressed genes between cell clusters reveals distinct transcriptional programs for seven clusters (Fig. 2C). Seven clusters of cell types were identified based on differential gene expression, referring to previous studies on muscle scRNA-seq [30–42], namely myocytes (*ACTN2*, *ACTN3*, and *MYO18B*), fibroblast/fibro/adipogenic progenitors (FAPs) (*PDGFRA*, *SMOC2*, *APOD*, and *GPX3*), satellite cells (*PAX7* and *TAGLN3*), endothelial cells (*CDH5*, *PECAM1*, and *VWF*), macrophage (*CD163*, *PTPRC*, and *SRGN*), pericytes (*RGS5*, *KCNJ8*, and *ACTA2*), and adipocytes (*ADIPOQ*, *DGAT2*, and *PLIN1*) (Fig. 2D). Gene expression profiles of markers used to identify cell types were showed in Fig. 2E. The cell differentiation status shows that the branch in the lower right corner of Fig. 2F is likely the origin of differentiation. Pseudotime analysis revealed the differentiation pathway of FAPs and satellite cells, suggesting their ability to differentiate into muscle cells (Fig. 2G).

### FAPs subpopulation analysis and DEGs enrichment pathways

**Fig. 3:**
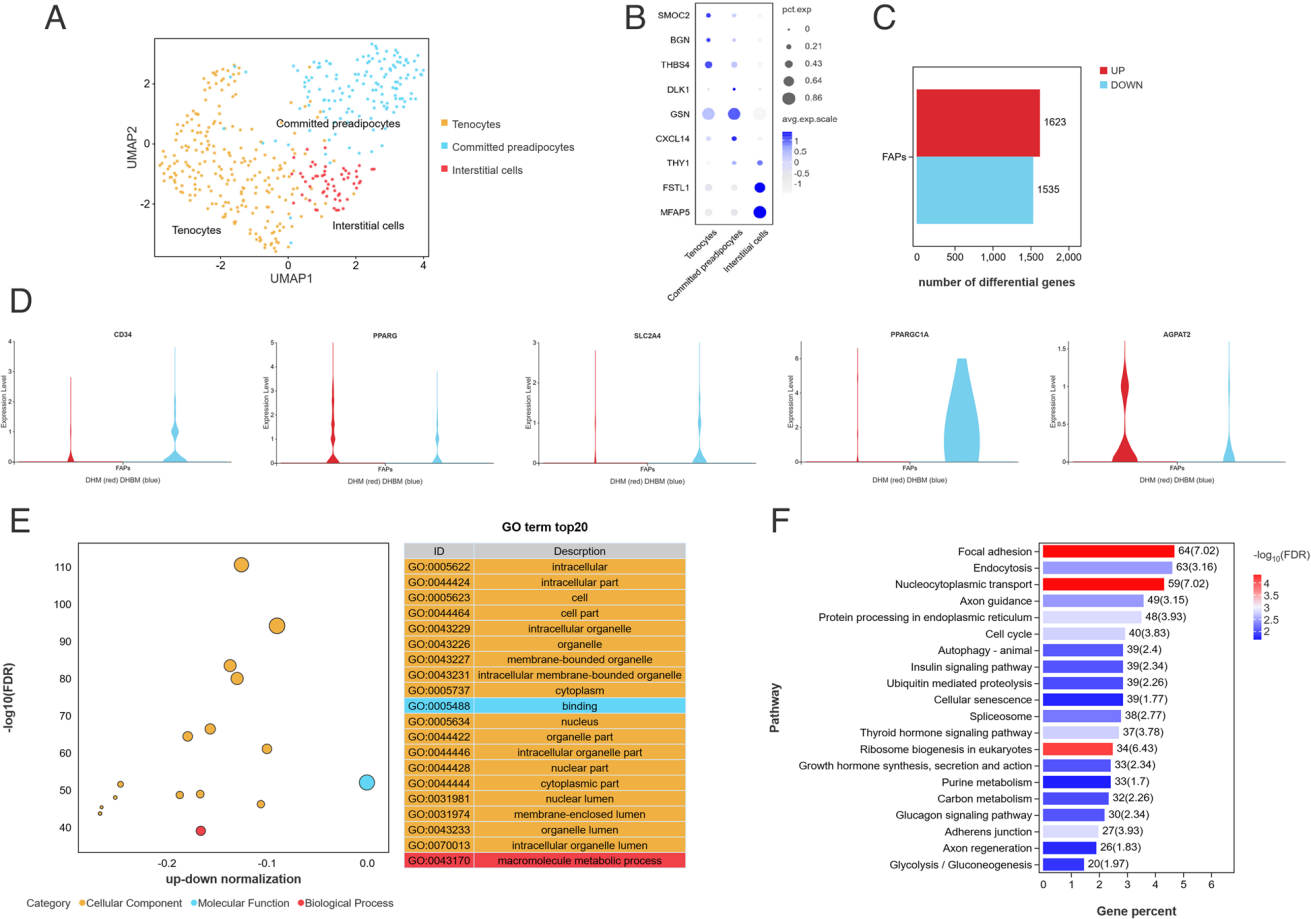
Further analysis of FAPs subpopulations and DEGs enrichment analysis. **A** UMAP diagram of three subclusters of FAPs. **B** Marker genes are used to distinguish the three subclusters. **C** The number of DEGs up-regulated/down-regulated by DHBM. **D** Changes in the expression levels of adipogenesis-related genes (*CD34*, *PPARG*, *SLC24A*, *PPARGC1A*, and *AGPAT2*) in FAPs of DHBM. **E** GO enrichment of DEGs between DHM and DHBM. The larger the bubble, the greater the number of DEGs. Yellow represents cellular components, blue represents molecular functions, and red represents biological processes. The significance of pathways enriched by DEGs is represented by the negative logarithm of the FDR value. **F** DEGs enrichment of KEGG pathways. The number on each entry represents the number of DEGs

FAPs cells in Dahe pig and Dahe black pig accounted for 15.94% and 11.27% of the total number of cells respectively (Table 1). Further analysis based on shared nearest neighbor clustering of FAPs subpopulations using the single-cell R tool package Seurat returned 3 subclusters (Fig. 3A). Referring to previous studies, the subcluster with high expression of *SMOC2*, *BGN*, and *THBS4* was defined as tenocytes; the subcluster with high expression of *DLK1*, *GSN*, and *CXCL14* was defined as committed preadipocytes; and the subcluster with high expression of *THY1*, *FSTL1*, and *MFAP5* was defined as interstitial cells (Fig. 3B). Compared to FAPs from DHM, DHBM had 1623 up-regulated DEGs and 1535 down-regulated DEGs (Fig. 3C). Among these genes, particular attention has been given to genes related to lipogenesis. Preadipocyte-enriched genes (*CD34*), the adipogenic master regulators (*PPARG*), late lipogenic genes (*SLC24A* and *PPARGC1A*), and lipogenic genes (*AGPAT2*) showed differential expression between DHM and DHBM. Among them, the expression levels of *CD34*, *SLC24A*, and *PPARGC1A* were significantly upregulated in DHBM, while the expression levels of *PPARG* and *AGPAT2* were significantly downregulated (*p* < 0.05) (Fig. 3D, Additional file 3). Based on the DEGs, the GO enrichment analysis of the top 20 pathways revealed that the FAPs subpopulations had the highest number of cellular component entries, with 18 entries. Additionally, 18 of the top 20 classes were accounted for cellular components, while two were accounted for the molecular function and biological process, respectively (Fig. 3E). The top 20 pathways enriched by KEGG were not associated with lipid metabolism, which suggests that the FAPs subpopulations may not have the ability to metabolize lipids (Fig. 3F).

**Table 1:** Cell numbers for 7 cell types

Cell type	DHM	DHBM
Myocytes	881 (64.12%)	1391 (73.60%)
FAPs	219 (15.94%)	213 (11.27%)
Satellite cells	51 (3.71%)	135 (7.14%)
Endothelial cells	92 (6.7%)	69 (3.65%)
Macrophage	79 (5.75%)	43 (2.28%)
Pericytes	46 (3.35%)	28 (1.48%)
Adipocytes	6 (0.44%)	11 (0.58%)

### Pathway enrichment analysis of DEGs in adipocyte subpopulations

**Fig. 4:**
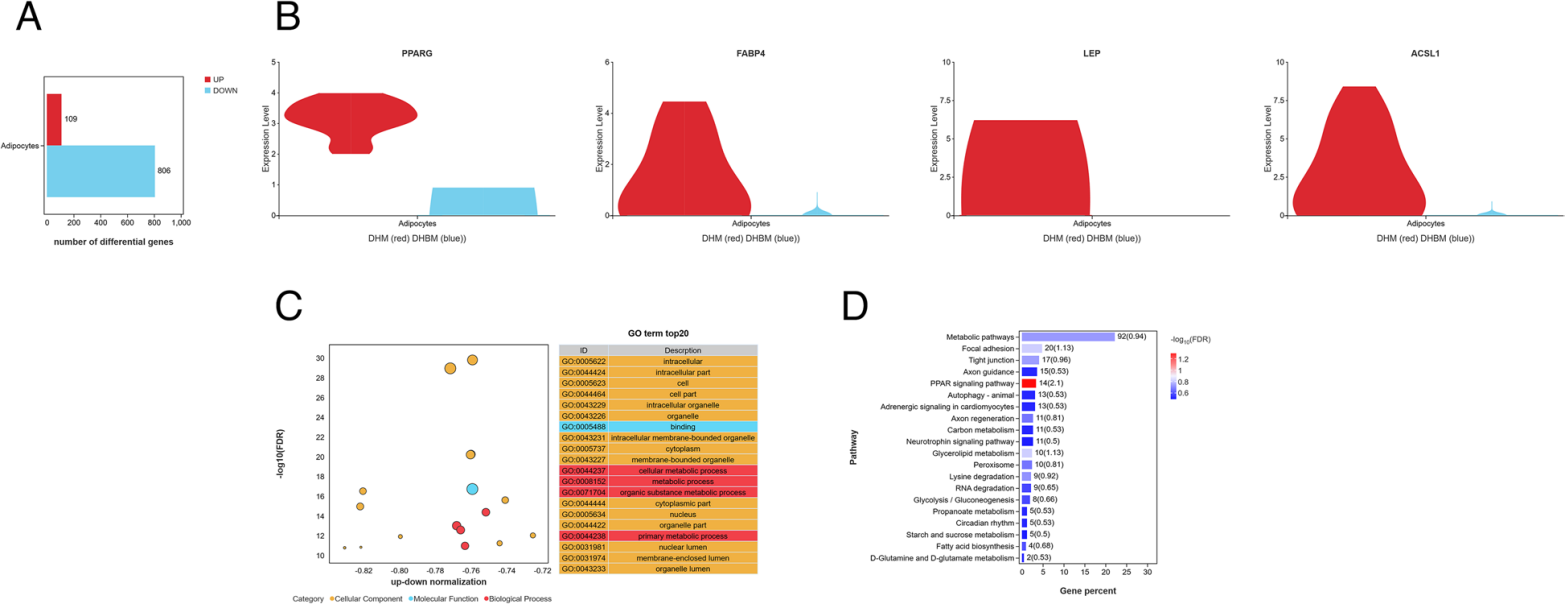
Further analysis of DEGs in adipocyte subpopulations. **A** The number of DEGs up-regulated/down-regulated by DHBM. **B** Characterization of adipogenesis-related DEGs (*PPARG*, *FABP4*, *LEP* and *ACSL1*) in DHBM adipocytes. **C** GO enrichment of DEGs between DHM and DHBM. (D) DEGs enrichment of KEGG pathways

Adipocyte cells in Dahe pig and Dahe black pig accounted for 0.44% and 0.58% of the total number of cells respectively (Table 1). The DHBM adipocyte subpopulation exhibited 806 down-regulated genes and only 109 up-regulated genes (Fig. 4A). The adipogenic master regulators (*PPARG*), late lipogenic genes (*FABP4* and *LEP*), and lipogenic genes (*ACSL1*) showed differential expression between DHM and DHBM. The expression of these four genes was lower in DHBM, but highly expressed in DHM (Fig. 4B, Additional file 3). In the FAPs subpopulation, the late lipogenic genes *SLC24A* and *PPARGC1A* were highly expressed in DHBM, but no differential expression of these genes was identified in the adipocyte subpopulation. This indicates that these two cell subpopulations have distinct transcriptional dynamics. In the GO enrichment analysis of the top 20, 15 pathways were related to cellular components, one pathway was associated with molecular function, and 4 pathways were linked to biological processes (Fig. 4C). KEGG enrichment analysis was performed on the DEGs. Pathways related to lipid metabolism were enriched, including the PPAR signaling pathway, glycerolipid metabolism, and FA biosynthesis (Fig. 4D).

### Overall lipid composition characteristics

**Fig. 5:**
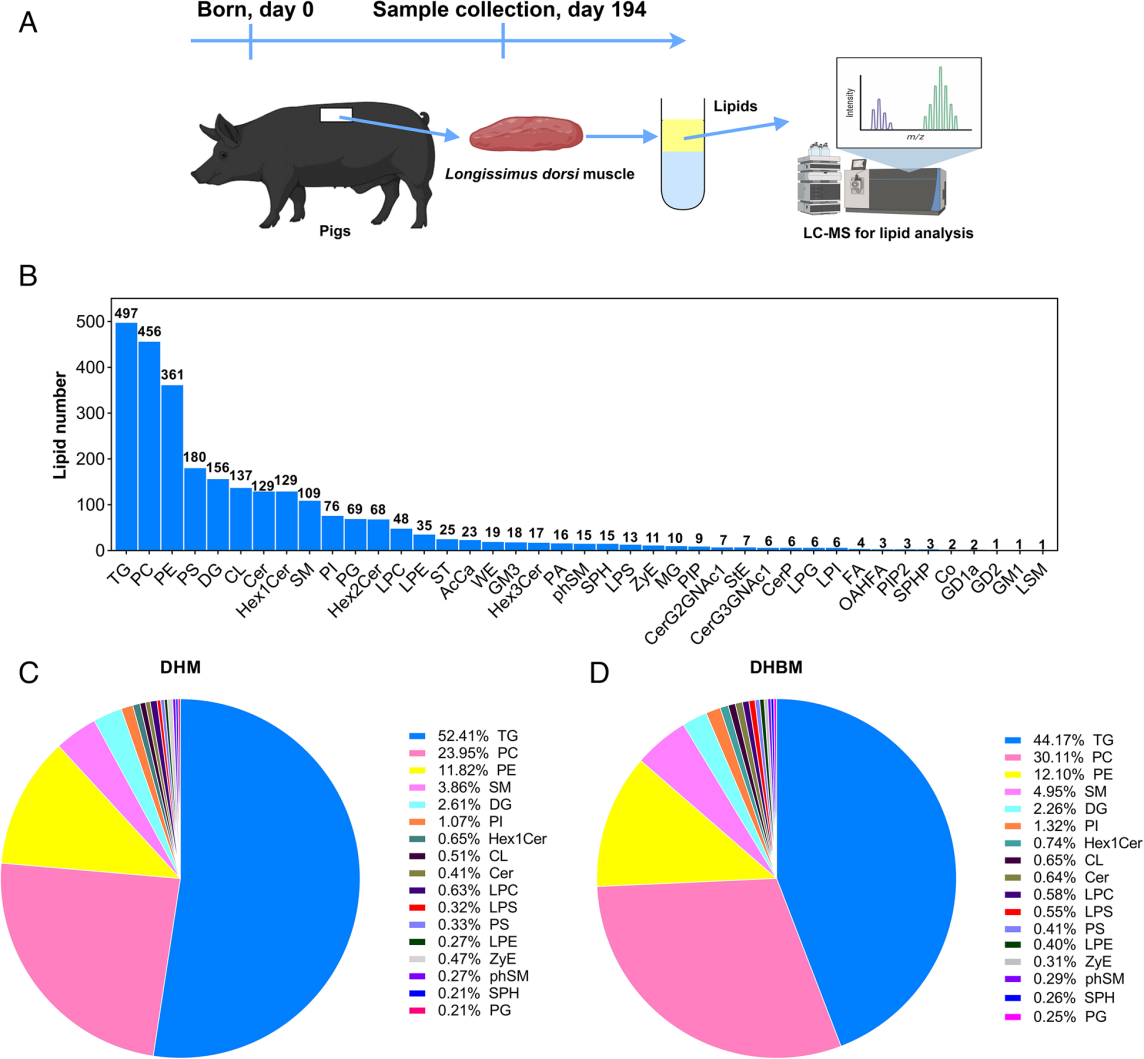
Characteristics of the overall lipid composition of the *longissimus dorsi* muscle of Dahe pigs and Dahe Black pigs. **A** Porcine *longissimus dorsi* muscle preparation and protocol for the lipidomic analysis. **B** Lipid classes were identified in both positive and negative ion modes, along with the number of lipid molecules in each class. CerG3GNAc1: simple Glc series, CerP: ceramides phosphate, CL: cardiolipin, Co: coenzyme, GD1a: gangliosides, GD2: gangliosides, GM1: gangliosides, GM3: gangliosides, Hex1Cer: simple Glc series, Hex2Cer: simple Glc series, Hex3Cer: simple Glc series, LPC: lysophosphatidylcholine, LPE: lysophosphatidylethanolamine, LPG: lysophosphatidylglycerol, LPI: lysophosphatidylinositol, LPS: lysophosphatidylserine, LSM: lysosphingomyelin, MG: monoglyceride, OAHFA: OAcyl-(gamma-hydroxy) FA, PA: phosphatidic acid, PG: phosphatidylglycerol, phSM: sphingomyelin, PIP: phosphatidylinositol, PIP2: phosphatidylinositol, PS: phosphatidylserine, SPH: sphingosine, SPHP: sphingosine phosphate, ST: sulfatide, StE: stigmasterol ester, WE: wax esters, ZyE: zymosterol ester. **C** In DHM, lipid classes account for more than 1% of all identified lipid classes and were utilized to generate pie charts. **D** In DHBM, lipid classes account for more than 1% of all identified lipid classes and were utilized to generate pie charts

To compare the lipid profile of the *longissimus dorsi* muscle in Dahe Black pigs with that of Dahe pigs, a lipidomic analysis method based on LC–MS was used to examine their characteristics (Fig. 5A). A total of 41 lipid classes and 2699 lipid species were identified in DHM and DHBM. The TG lipid class contains 497 lipid species, the highest number among the 41 lipid classes, followed by phosphatidylcholine (PC), which contains 456 lipid species (Fig. 5B). The lipid classes of DHM and DHBM, accounting for more than 1%, include TG (52.41% and 44.17%), PC (23.95% and 30.11%), phosphatidylethanolamine (PE) (11.82% and 12.10%), phSM (3.86% and 4.95%), diglyceride (DG) (2.61% and 2.26%), and PI (1.07% and 1.32%) (Fig. 5C and D).

### Changes in the overall lipid class between Dahe pigs and Dahe Black pigs

**Fig. 6:**
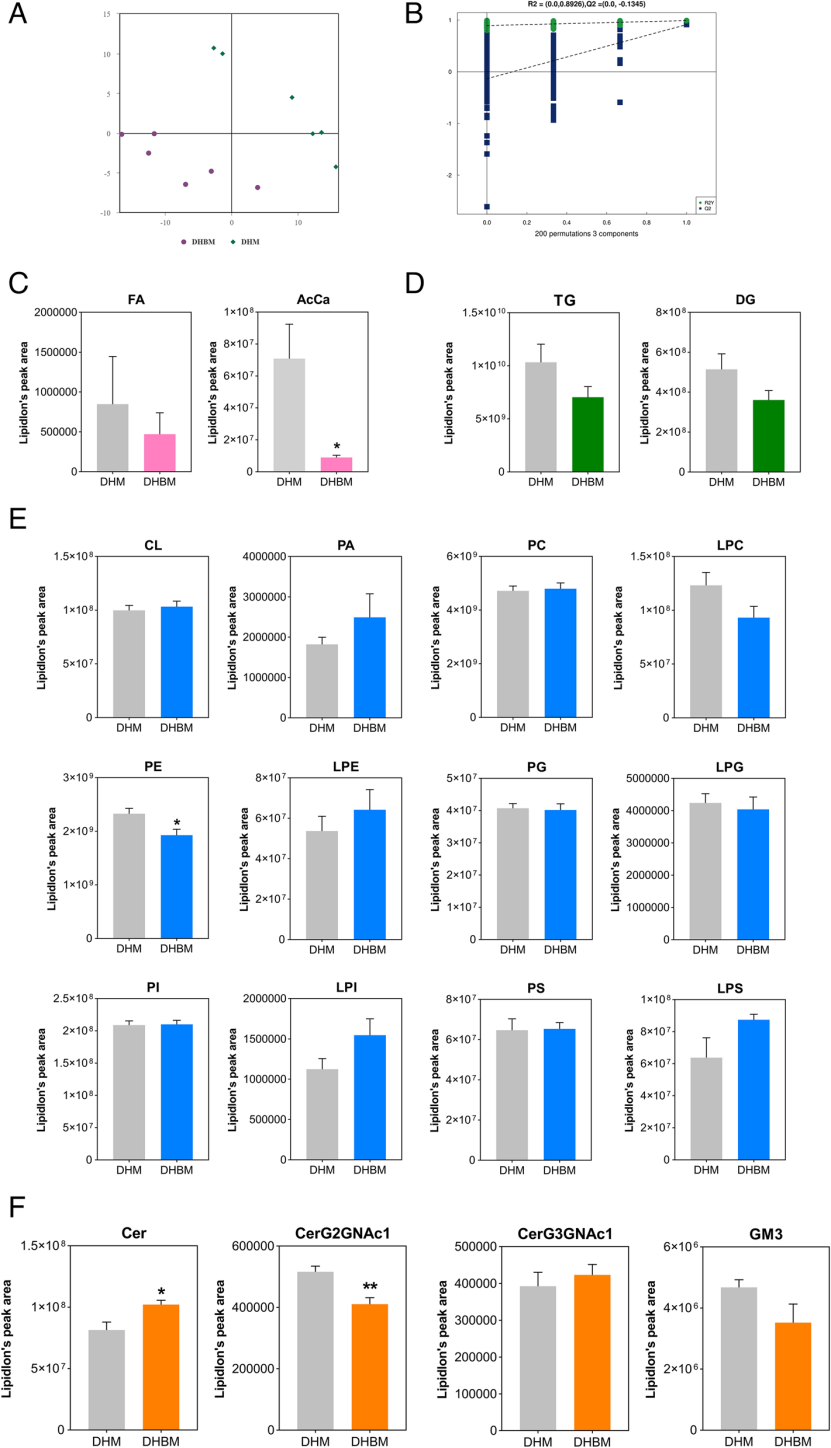
Differences in lipid composition of *longissimus dorsi* muscle between Dahe pigs and Dahe Black pigs. **A** A PLS-DA model was constructed using DHM and DHBM samples to show group differences. **B** Permutation test on PLS-DA model. Lipidlon’s peak area change of (**C**) fatty acyls (FA and AcCa: acyl carnitine), (**D**) glycolipids (TG and DG: diglyceride), (**E**) phospholipids (CL and PA, PC, LPC, PE, LPE, PG, LPG, PI: phosphatidylinositol, LPI, PS, and LPS) and (**F**) sphingolipids (Cer and CerG2GNAc1: simple Glc series, CerG3GNAc1: simple Glc series, and GM3: gangliosides) in DHM vs DHBM. *p < 0.05, **p < 0.01

Partial least squares regression was used to establish a relationship model between the relative expression of lipids and the samples (Fig. 6A). After conducting sevenfold cross-validation, the replacement retention gradually decreases, leading to a gradual decrease in both R2 and Q2 of the random model (Fig. 6B). This indicates that the model does not exhibit overfitting and that the difference between DHM and DHBM is valid. The PLS-DA model can differentiate between the DHM and DHBM groups. The levels of FA and acyl carnitine (AcCa) in DHBM were lower than those in DHM, with AcCa reaching a significant level (*p* < 0.05) (Fig. 6C). The levels of TG and DG in DHBM were not significantly different from those in DHM (Fig. 6D). There was a significant decrease in the relative abundance of PE in DHBM (*p* < 0.05) (Fig. 6E). Sphingolipids of DHBM, including ceramides (Cer) and simple Glc series (CerG2GNAc1), showed significant changes, with increased levels of Cer and decreased levels of CerG2GNAc1 (*p* < 0.05) (Fig. 6F).

### Differential changes in lipid species in pigs and correlation analysis

**Fig. 7:**
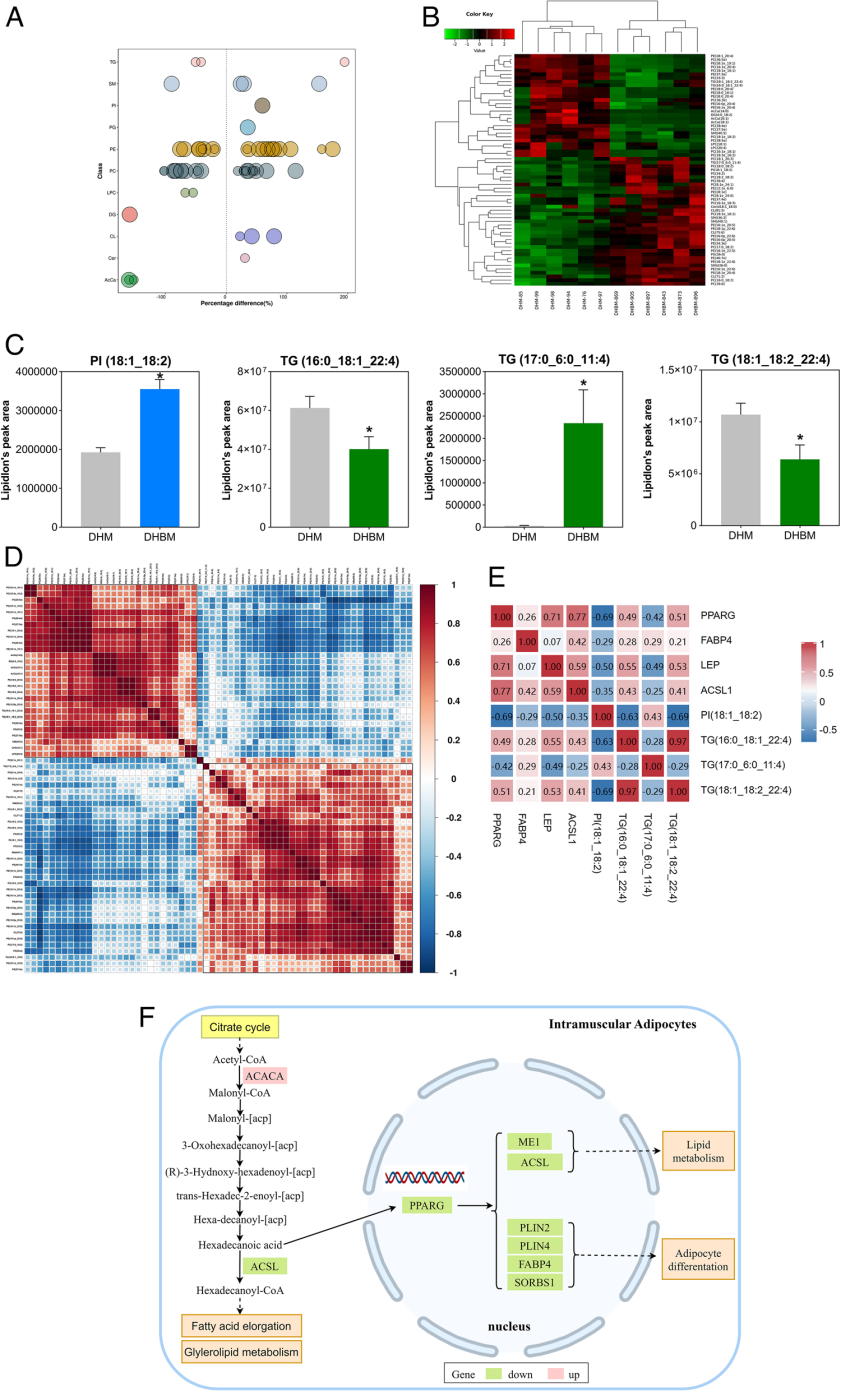
Differential lipid species of *longissimus dorsi* muscle between Dahe pigs and Dahe black pigs. **A** Lipid molecules with significant differences (Variable important in projection, VIP > 1, *p* < 0.05) are displayed as bubble plots. The ordinate represents each lipid class, distinguished by different colors. Each bubble represents a different lipid species, and the size of the bubble indicates the extent of the difference. **B** Clustering heatmap of differential lipid species. Each row represents a distinct lipid species, and each column represents a sample. Red represents relatively high expression, while green represents relatively low expression. Named according to the International Lipid Classification and Nomenclature Committee. Lipids are divided into 8 types: Glycerophospholipids, Sphingolipids, Glycerolipids, Sterol lipids, Prenol lipids, Fatty Acyls, Glucosylsphingosine, and Polyketides. Among them, each type is divided into different "classes" based on various polar groups. Each "class" is then divided into different "species" based on the number of carbon atoms in the carbon chain and the number of double bonds. For example, TG (17:0_6:0_11:4) indicates that this triglyceride contains three fatty acyl groups, namely 17:0, 6:0, and 11:4 fatty acids. **C** All lipid species with significant differences in the PI and TG categories were identified using VIP > 1 and *p < 0.05. **D** Pearson correlation analysis of differential lipid species. Red indicates a positive correlation, while blue indicates a negative correlation. The color becomes more intense as the correlation coefficient increases. **E** Spearman correlation between differential lipid species and differential genes related to lipogenesis in adipocytes. Red indicates a positive correlation, while blue indicates a negative correlation. *p < 0.05, **p < 0.01. **F** Transcriptional patterns of lipid metabolism-related genes in the longissimus dorsi muscle of Dahe Black pigs

All lipid species with significant differences between DHM and DHBM were visualized using bubble plots (Fig. 7A). A total of 63 different lipid species were identified. Among them, the relative expression levels of 35 lipid species in DHBM were higher than those in DHM, while 28 were lower than those in DHM (Fig. 7B and Additional file 4). There was a significant increase in the TG fatty acyl chains C6:0, C17:0, and C11:4, and a significant decrease in C16:0, C18:1, C18:2, and C22:4 in DHBM (*p* < 0.05) (Fig. 7C). Although there was no significant difference in PI between the two groups, DHBM showed a significant increase in acyl fatty chains C18:1 and C18:2 within this category (Figs. 6F and 7C). There is a significantly positive correlation between PI (18:1_18:2) and TG (17:0_6:0_11:4) (*p* < 0.05), but an extreme significantly negative correlation with TG (16:0_18:1_22:4) and TG (18:1_18:2_22:4) (*p* < 0.01) (Fig. 7D and Additional file 5). There is a significant negative correlation between PI (18:1_18:2) and *PPARG* (*p* < 0.05) (Fig. 7E). In addition, the higher expression of *ACACA* in DHBM increased the C16:0 (Hexadecanoic acid), which may be a ligand that down-regulates the expression of *PPARG*, resulting in down-regulation of genes expression associated with lipogenesis and FA transport (Fig. 7F).

## Discussion

The process of lipid deposition in pigs is mainly reflected in the middle and late stages of growth. Muscle and bone develop first, with muscles growing relatively quickly and bones relatively slowly, and when fat formation accelerates during the later growth stages, the rates of muscle and bone deposition decline [43]. A small effect of body weight on the lipid content of adipose tissue was observed when comparing pigs weighing 105 kg, 120 kg, and 135 kg [44]. Our team has conducted a lot of research on fat deposition in Wujin pigs. Among them, the average daily weight gain of Wujin pigs was lower than that of Landrace pigs, while the fat cell diameter and IMF content were higher [45]. IMF content increased with feeding time. The EE contents of the longissimus dorsi muscle of DHM and DHBM were 7.98% and 3.30% (Fig. 1 and Additional file 1). IMF content is considered crucial for improving meat quality, and consumer acceptance is higher when IMF content falls within the range of 2.2% to 3.4% [46, 47]. Mature muscle fibers are multinucleated cells that predominate in skeletal muscle tissue. However, due to limitations in cell isolation methods and sequencing on cell diameter, only a small portion of the cells can be captured in single-cell studies [48]. Therefore, this study used snRNA-seq to analyze the heterogeneity of *longissimus dorsi* muscle cells in Dahe pigs and Dahe black pigs. The analysis of the expression patterns of DEGs related to FA synthesis, such as *ACACA*, *ACSF3*, *OXSM*, *CBR4*, and *HSD17B8*, between Mashen pigs and Large White pigs showed that IMF accumulation occurred between 90 and 180 days [49]. The key genes that regulate IMF deposition are *ADIPOQ*, *CIDEC*, *CYP4B1*, *DGAT2*, *LEP*, *OPRL1*, *PLIN1*, *SCD* and *THRSP* [50]. The *GPAT1*, *AGPAT1*, *AGPAT2*, *LIPIN1*, and *DGAT* genes are related to TG biosynthesis [51].

In FAPs subpopulations of Dahe black pig, the expression levels of *CD34*, *SLC24A*, and *PPARGC1A* were significantly upregulated, while the expression levels of *PPARG* and *AGPAT2* were significantly downregulated (Fig. 3). *CD34* is a marker for vascular endothelial cells, and a larger vascular network is associated with increased IMF content [52]. *PPARGC1A* plays a crucial role in reducing lipid deposition [53]. *UBC*, *SLC27A5*, *RXRG*, *PRKCQ*, *PRKAG2*, *PPARGC1A*, *PLIN5*, *PLIN4*, *IRS2*, and *CPT1B* play a role in regulating the growth and development of the *longissimus dorsi* muscle in Ningxiang pigs [54]. The genes *PPARA*, *PPARG*, *SREBF1*, and *PPARGC1A* genes are involved in regulating fat deposition in the *longissimus dorsi* muscle of pigs. Among these genes, *PPARGC1A* shows an imbalance in allele-specific expression in Polish Large White pigs, Polish Landrace pigs, and Pietrain pigs [55]. The *PPARGC1A* gene encodes peroxisome proliferator-activated receptor coactivator 1α, which coactivates transcription factors that control skeletal muscle fiber type switching and skeletal muscle fiber formation [56]. It could promote mitochondrial biogenesis and regulate skeletal muscle metabolism by mediating glycolysis and the TCA cycle, promoting intramuscular FA oxidation, and driving the transformation of fast muscle fibers into slow muscle fibers [57]. The *PPARGC1A* gene was significantly overexpressed in the FAPs cell subpopulation of Dahe black pigs, and there was significant enrichment in the glycolysis/gluconeogenesis pathway (Fig. 3). This suggests that it may be a crucial regulatory gene influencing the fat deposition between Dahe pigs and Dahe black pigs.

There was a significant decrease in the expression of adipogenic master regulators (*PPARG*), late lipogenic genes (*FABP4* and *LEP*), and lipogenic genes (*ACSL1*) in Dahe black pig (Fig. 4). Intramuscular preadipocytes could differentiate into mature adipocytes in vitro, and there is differential expression of differentiation-related genes such as *PPARG*, *CEBPA*, *FASN*, and *SREBF1* between Wujin and Landrace pigs [58]. There is a positive correlation between IMF deposition and *PPARG* mRNA expression in the *longissimus dorsi* muscle of Erhualian pigs native to China [59]. The IMF content and expression of adipogenesis-related genes, such as *C/EBPα*, *FABP4*, and *SCD1*, were higher in Qingyu pigs than in Yorkshire pigs [60]. The *FABP4* gene is involved in the regulation of IMF deposition in Duroc pigs [61]. The genes *ARID5B*, *CPT1B*, *ACSL1*, *LPIN1*, *HSP90AA1*, *IRS1*, *IRS2*, *PIK3CA*, *PIK3CB*, and *PLIN2* in the *longissimus dorsi* muscle of Songliao black pigs and Landrace pigs may play a crucial role in IMF deposition [62]. The *ACSL1* gene is associated with the biological process of long-chain FA import and signal transduction, and the protein it encodes is involved in synthesizing long-chain acyl-CoA esters, FA degradation, and phospholipid remodeling [63]. Excessive fat accumulation and increased IMF mass in pigs may be related to increased *LEP* gene expression, and obese individuals were found to be resistant to leptin [64]. The EE content of the *longissimus dorsi* muscle in Dahe pigs is higher than that in Dahe black pigs, while the CP content is lower. Increasing muscularity will also dilute the final fat content of muscle [65]. Adipocyte cells in Dahe pig and Dahe black pig accounted for 0.44% and 0.58% of the total number of cells respectively. Adiposity in the pig was due to cellular hypertrophy rather than cellular hyperplasia, since during growth, the leaner conventional pigs (30.6% extramuscular fat) contained more adipose cells than the fatter pigs (46.6% extramuscular fat) [66]. Although Dahe pigs had lower numbers of adipocytes, they were more active in lipogenesis, which may be the reason for their higher IMF content.

The DEGs of adipocyte subpopulations in Dahe pigs and Dahe black pigs were enriched in the PPAR signaling pathway (Fig. 4). Adipogenesis is driven by the PPAR signaling pathway, which enhances angiogenesis, lipid metabolism, migration, and tumorigenesis capabilities [67]. Muscle-specific overexpression of PPARG was found to promote fat deposition by activating adipocyte differentiation regulatory factors such as FABP4 and CCAAT enhancer-binding protein, while enhancing the expression of *LPL*, *FABP4*, and *PLIN1* [68]. Transcriptome analysis of the *longissimus dorsi* muscle tissue of Huai pigs revealed that DEGs were mainly involved in pathways related to amino acid metabolism, lipid metabolism, and PPAR signaling pathways [69]. The transcriptome analysis of the *longissimus dorsi* muscle of Anqing Six-end-white pigs (with high and low IMF) showed enrichment of DEGs related to lipid metabolism, lipid biosynthesis, and the PPAR signaling pathway [70]. Another study of Diannan small-ear pigs revealed an upregulation of genes associated with the PPAR signaling pathway, FA metabolism, and oxidative phosphorylation processes, which may be linked to IMF deposition [71].

Changes in the expression levels of lipid metabolism-related genes in the transcriptome analysis affect lipid composition. The relative abundance of TG, DG, PE, CerG2GNAc1 and GM3 in Dahe pigs was higher than that in Dahe black pigs (Figs. 5 and 6). Lipidomic analysis of Xidu black pigs showed that saturated FAs, PI, and PS may contribute to IMF deposition [72]. The rate of fat droplet formation in Laiwu pigs is rapid, and the triglyceride content is higher than that of Yorkshire pigs, corresponding to its higher IMF content [73]. There are differences in carnitine, DG, TG, phSM, CL, FA, PC, and PE between Jianhe Baixiang pigs and Large White pigs. Jianhe Baixiang pigs have higher levels of PC and PE double bond substances [74]. Non-targeted lipidomic analysis of Laiwu black pig muscle showed higher IMF and TG content compared to Duroc pig × (Landrace pig × Yorkshire pig) and Beijing Heiliu pigs, and the LPC content was lower [75]. The lipidomics analysis of Laiwu pigs with high and low fat content revealed that those with high fat content exhibited increased levels of TG, DG, MG, and monohexose ceramide in their lipid composition [76]. There was a significant increase in the TG fatty acyl chains C6:0, C17:0, and C11:4, and a significant decrease in C16:0, C18:1, C18:2, and C22:4. The levels of C16:0 and C18:1 FA in the *longissimus dorsi* muscle of Dahe pigs were significantly higher than those in Dahe black pigs [77]. This shows that Dahe pigs and Dahe black pigs have distinct lipid composition characteristics, and pigs with high IMF content tend to have more glycerolipids. In overall, the lipid classes were similar in Dahe pigs and Dahe black pigs. This indicates that the transcriptomic differences promote fat accretion (fat content) rather than fat modification (fatty acid elongation, desaturation, etc.).

The lipid composition of the body is regulated by multiple genes, such as *ACSL*, *FABP*, and *PPAR*. Exogenous FAs increase the amount of newly synthesized PI through *ACSL4* overexpression [78]. *ACSL4* is a unique isozyme that preferentially catalyzes several polyunsaturated FAs such as C20:4 [79]. The expression of adipogenic differentiation marker genes *PPARG* and *CEBPA*, as well as lipid anabolism marker genes *ACC*, *FASN*, *SCD1*, *SREBP1*, *FABP4*, *ACSL1*, *LPL*, and *DGAT1*, was inhibited. This led to a reduction in TG content and inhibited the production of lipid droplets in bovine adipocytes [80]. Both the size of adipocytes and the recruitment of new adipocytes are controlled by *FABP4* [81]. An in vitro study demonstrated that elevating the concentration of C18:0 FA increased lipid accumulation in preadipocytes and raised *FABP4* expression levels [82]. Cells cultured with excess C18:0 FA exhibit a higher rate of lipid accumulation, and C18:0 FA can stimulate the expression of *C/EBPA* and *PPARG* [83]. C16:0 and C18:0 FA treatments increased *PPARG* expression in croaker liver [84]. In this study, C16:0 FA may downregulate the expression level of *PPARG* gene, which leads to the downregulation of fat metabolism-related genes such as *ACSL*, *PLIN2*, and *FABP4* in DHBM compared with DHM (Fig. 7). This may suggest that the lipid deposition ability of Dahe pigs is stronger than that of Dahe black pigs.

## Conclusions

In conclusion, the transcriptomic profiles and lipid composition characteristics of the *longissimus dorsi* muscle in Dahe pigs and Dahe black pigs have been provided. Interestingly, the lipid metabolism pathway was not included in the top 20 enriched KEGG pathways of FAPs. In contrast, lipid metabolism pathways such as the PPAR signaling pathway and FA biosynthesis were enriched in adipocytes. C16:0 FA may downregulate the expression level of *PPARG* gene, which leads to the downregulation of fat metabolism-related genes such as *ACSL*, *PLIN2*, and *FABP4* in DHBM compared with DHM. This may suggest that the lipid deposition ability of Dahe pigs is stronger than that of Dahe black pigs. These findings could provide a foundation for genetically improving and depositing IMF at the cellular level.

### Supplementary Information

Supplementary Material 1.

Supplementary Material 2.

Supplementary Material 3.

Supplementary Material 4.

Supplementary Material 5.

## Data Availability

Data is contained within the article or supplementary material. Additional data that support the findings of this study are available from the corresponding author upon reasonable request. The single nucleus RNA sequencing data used in this study is deposited in NCBI databases under accession number: PRJNA1113324. SRA database accession number: SRR29079131 and SRR29079130.

## Abbreviations

AcCa: Acyl carnitine
Cer: Ceramides
CerG2GNAc1: Simple Glc series
CerG3GNAc1: Simple Glc series
CerP: Ceramides phosphate
CL: Cardiolipin
Co: Coenzyme
CP: Crude protein
DEGs: Differentially expressed genes
DG: Diglyceride
EE: Ether extracts
FA: Fatty acid
FAPs: Fibroblast/fibro/adipogenic progenitors
GD1a: Gangliosides
GD2: Gangliosides
GEM: Gel bead-in-EMlusion
GM1: Gangliosides
GM3: Gangliosides
GO: Gene Ontology
Hex1Cer: Simple Glc series
Hex2Cer: Simple Glc series
Hex3Cer: Simple Glc series
IMF: Intramuscular fat
KEGG: Kyoto Encyclopedia of Genes and Genomes
LPC: Lysophosphatidylcholine
LPE: Lysophosphatidylethanolamine
LPG: Lysophosphatidylglycerol
LPI: Lysophosphatidylinositol
LPS: Lysophosphatidylserine
LSM: Lysosphingomyelin
MG: Monoglyceride
MTBE: Methyl tert-butyl ether
OAHFA: OAcyl-(gamma-hydroxy) FA
PA: Phosphatidic acid
PC: Phosphatidylcholine
PE: Phosphatidylethanolamine
PG: Phosphatidylglycerol
phSM: Sphingomyelin
PI: Phosphatidylinositols
PIP: Phosphatidylinositol
PIP2: Phosphatidylinositol
PLS-DA: Partial least squares discriminant analysis
PS: Phosphatidylserine
scRNA-seq: Single cell transcriptome sequencing
snRNA-seq: Single-nucleus RNA sequencing
SPH: Sphingosine
SPHP: Sphingosine phosphate
ST: Sulfatide
StE: Stigmasterol ester
TG: Triglycerides
UMAP: Uniform manifold approximation and projection
UMI: Unique molecular identifier
VIP: Variable important in projection
WE: Wax esters
ZyE: Zymosterol ester
